# DECLINING HEALTH AND SENSE OF MASTERY CAN EXPLAIN THE INVERSE RELATION BETWEEN OLDER AGE AND WISDOM

**DOI:** 10.1093/geroni/igad104.0397

**Published:** 2023-12-21

**Authors:** Monika Ardelt, Dilip Jeste

**Affiliations:** University of Florida, Gainesville, Florida, United States; University of California, San Diego, San Diego, California, United States

## Abstract

Previous research found an inverse U-shaped association between age and three-dimensional wisdom with the apex in late midlife. What might explain the negative relation between age and wisdom during the later years of life? We investigated the impact of physical health and mastery, using data from the Successful AGing Evaluation (SAGE) study of 976 adults between the ages of 51 and 99 years (M = 77.23, SD = 12.18). Physical health tends to decline with advancing years and, therefore, might reduce older adults’ sense of mastery and control over their life. Ill health and feelings of diminished control, in turn, might lead older adults to focus on improving their health and regaining control and reduce their desire to comprehend the deeper truth about life (cognitive wisdom dimension), look at things from different perspectives (reflective wisdom dimension), and care about others (compassionate wisdom dimension). As expected, results showed that age correlated negatively with subjective health, wisdom, and mastery, while health, wisdom, and mastery were positively correlated. In multivariate regression analyses, age and ill health remained negatively related to mastery even after controlling for education, gender, and race and the positive association with wisdom. By contrast, the negative relations of age and ill health on wisdom became non-significant after mastery was entered into the model. If wisdom declines with age in the later years of life due to ill health that results in a perceived loss of mastery, enabling older adults to maintain their sense of control might offset this decline.

